# Comparative analysis of motor skill acquisition in a novel bimanual task: the role of mental representation and sensorimotor feedback

**DOI:** 10.3389/fnhum.2024.1425090

**Published:** 2024-09-11

**Authors:** Miguel Cienfuegos, Abdeldjallil Naceri, Jonathan Maycock, Risto Kõiva, Helge Ritter, Thomas Schack

**Affiliations:** ^1^Neurocognition and Action-Biomechanics Group, Bielefeld University, Bielefeld, Germany; ^2^Center for Cognitive Interaction Technology (CITEC), Bielefeld University, Bielefeld, Germany; ^3^Munich School of Robotics and Machine Intelligence (MSRM), Technical University of Munich (TUM), Munich, Germany; ^4^Margin UG, Bielefeld, Germany; ^5^Neuroinformatics Group, Bielefeld University, Bielefeld, Germany

**Keywords:** bimanual motor learning, maze, SDA-M, skill acquisition, biomechanics, tactile pressure, cognitive primitives

## Abstract

**Introduction:**

This study investigates the multifaceted nature of motor learning in a complex bimanual task by examining the interplay between mental representation structures, biomechanics, tactile pressure, and performance. We developed a novel maze game requiring participants to maneuver a rolling sphere through a maze, exemplifying complex sequential coordination of vision and haptic control using both hands. A key component of this study is the introduction of cognitive primitives, fundamental units of cognitive and motor actions that represent specific movement patterns and strategies.

**Methods:**

Participants were divided into two groups based on initial performance: poor performers (PPG) and good performers (GPG). The experimental setup employed motion capture and innovative tactile sensors to capture a detailed multimodal picture of the interaction process. Our primary aims were to (1) assess the effects of daily practice on task performance, biomechanics, and tactile pressure, (2) examine the relationship between changes in mental representation structures and skill performance, and (3) explore the interplay between biomechanics, tactile pressure, and cognitive representation in motor learning.

**Results:**

Performance analysis showed that motor skills improved with practice, with the GPG outperforming the PPG in maze navigation efficiency. Biomechanical analysis revealed that the GPG demonstrated superior movement strategies, as indicated by higher peak velocities and fewer velocity peaks during task execution. Tactile feedback analysis showed that GPG participants applied more precise and focused pressure with their right-hand thumb, suggesting enhanced motor control. Cognitively, both groups refined their mental representation structures over time, but the GPG exhibited a more structured and sophisticated cognitive mapping of the task post-practice.

**Discussion:**

The findings highlight the intertwined nature of biomechanical control, tactile feedback, and cognitive processing in motor skill acquisition. The results support established theories, such as the cognitive action architecture approach, emphasizing the role of mental representation in planning and executing motor actions. The integration of cognitive primitives in our analysis provides a theoretical framework that connects observable behaviors to underlying cognitive strategies, enhancing the understanding of motor learning across various contexts. Our study underscores the necessity of a holistic approach to motor learning research, recognizing the complex interaction between cognitive and motor processes in skill acquisition.

## 1 Introduction

Motor learning, the process of acquiring and refining motor skills through practice, is a fundamental aspect of human development, adaptation, and performance in various domains such as sports, rehabilitation, and daily activities (Newell, 1991). To design effective interventions and tailored training strategies for optimizing skill acquisition and performance, it is essential to have a comprehensive understanding of the underlying mechanisms of motor learning (Wulf and Shea, 2002; Wolpert et al., 2011). Although research in motor learning has traditionally focused on unimanual tasks or relatively simple bimanual tasks, the processes involved in more complex, naturalistic bimanual motor learning remain relatively unexplored (Swinnen, 2002; Swinnen and Wenderoth, 2004; Johansson and Flanagan, 2009).

**Figure 1:**
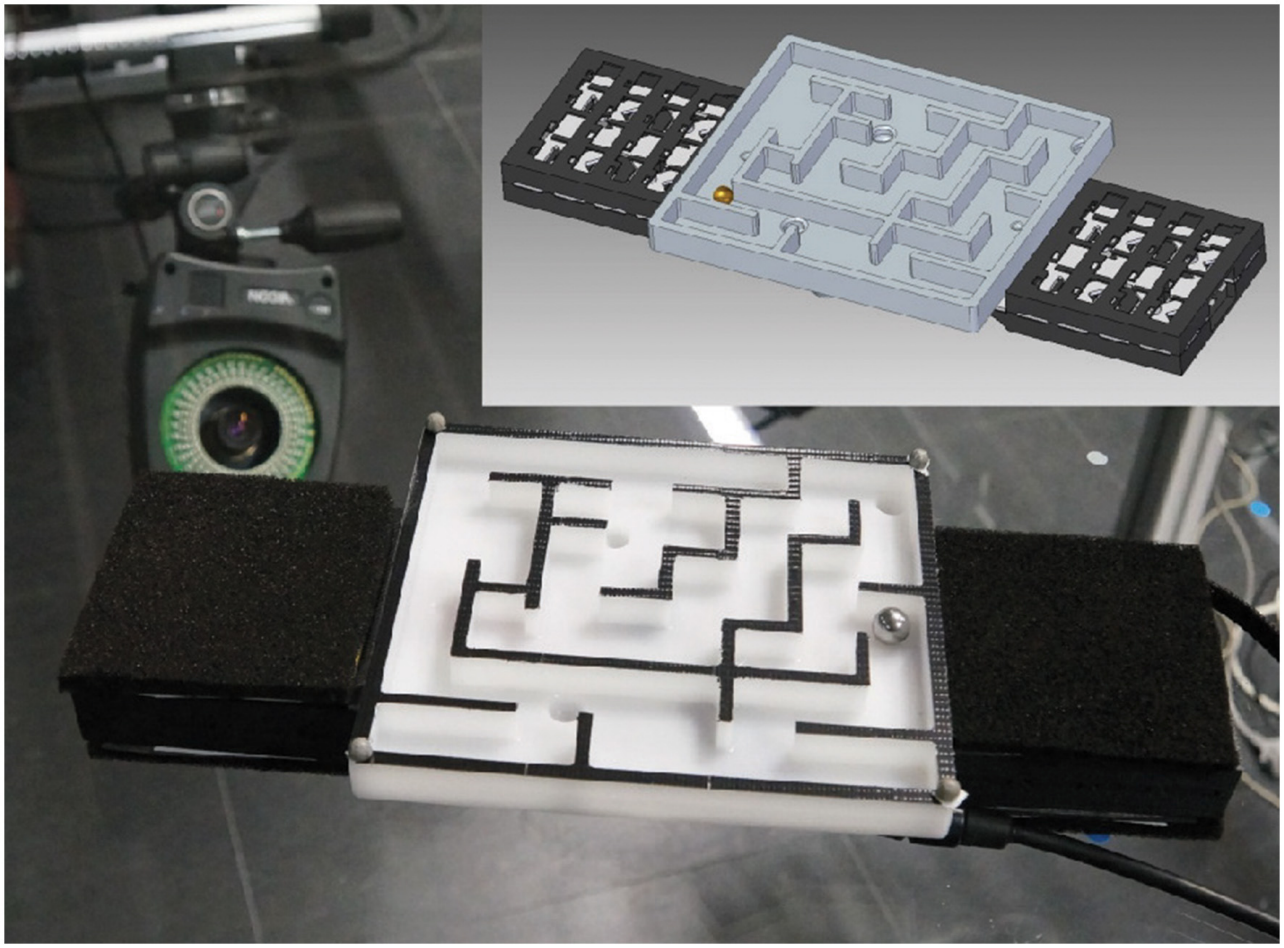
The figure illustrates the computer-aided design (CAD) of the maze game alongside the physical maze. The CAD design depicts the maze's structure, corridors, and walls, while the physical maze object showcases the tangible representation. The tactile sensors are visibly attached to the sides of the maze (Cienfuegos, 2024).

In this study, we aim to investigate how humans acquire a new manual skill that requires the organization of a sequence of rapid sensorimotor actions, each characterized by delicate coordination of tactile, kinesthetic, and visual sensing. Johannson and Flanaghan (Johansson and Flanagan, 2009) described manipulation tasks as a series of specific sensory events linked to subgoals. To achieve these subgoals, the brain has to select and execute appropriate action-phase controllers (Johansson and Flanagan, 2008). We have developed a task that involves maneuvering a rolling sphere through a configuration of “obstacles” forming a “maze,” held and controlled bi-manually (see Figure 1). This task exemplifies one of the numerous skills requiring complex sequential coordination of vision and haptic control through the use of two hands (Johansson and Flanagan, 2009; Bentivegna et al., 2003).

Our chosen platform's unique feature is the dynamic yet constrained nature of the maze game itself, combined with motion capture of the maze and the sphere and novel tactile sensors (Schürmann et al., 2011). These elements allow us to monitor the finger forces where the hands contact the maze to control its motion (Maycock et al., 2010) and provide a precise multimodal picture of the entire interaction process. Consequently, we can analyze and model the task's essential aspects and the progression of the underlying multimodal interaction patterns during learning. By devising a maze game task that exemplifies complex sequential coordination of vision and haptic control by using two hands (Kelso, 1984), combining motion capture and novel tactile sensors, we aim to obtain a precise multimodal picture of the entire interaction process. This enables us to analyze and model the essential aspects of the task and the progression of the underlying multimodal interaction patterns during learning (Atkeson and Hollerbach, 1985).

Moving the sphere through the maze can be viewed as a balanced compromise between simplifying the control task at the sensorimotor level (e.g., reducing the dimensionality of the control task when sliding the sphere along a maze wall toward a stable state instead of fully controlling it “in the open”) and simplifying the cognitive task at the planning/sequencing level (e.g., preferring a more straightforward path shape over a more complex one). Indeed, on the path toward complete control of the sphere in the open, we expect interesting intermediate steps such as navigating the sphere through the maze using a series of bounces off walls. We observed that learning involves phenomena characterized by different time scales, such as the transition from totally untrained, inferior initial performance to “mediocre task mastery” within a small number of trials (van Beers, 2009). We will characterize this rapid learning and compare it with the structure of the more prolonged (requiring multiple sessions) transition to “mastery.”

Daily practice significantly improves motor learning performance, enhancing movement accuracy and reducing error rates (Ericsson et al., 1993; Ericsson, 2008; Hardwick et al., 2017; Magill Richard and Anderson, 2021). This improvement has been suggested to be linked to biomechanical adaptations, resulting in more efficient movement patterns and optimized joint torques, particularly in lower limbs during activities like sit-to-stand transitions (Serbest et al., 2015; Schmidt et al., 2018). Practice also leads to behavioral changes, increasing speed, reducing cognitive load, and promoting automaticity (Haith and Krakauer, 2018; Krakauer et al., 2019). These changes are supported by neural adaptations, with shifts in brain activation patterns indicating reduced prefrontal cortex activity and increased subcortical engagement as tasks become more automatic (Poldrack et al., 2005; Sadtler et al., 2014). Tactile feedback plays a crucial role, allowing fine-tuning of motor actions based on sensory input, although specific studies on tactile pressure are limited (Johansson and Flanagan, 2009; Lederman and Klatzky, 2009; Gopaul et al., 2023). The interaction between these dimensions is vital; intrinsic feedback mechanisms are known to enhance motor learning (Wulf et al., 2010), while the importance of sensorimotor integration is well-documented (Wolpert et al., 1995; Körding and Wolpert, 2004; Ernst, 2007). Overall, daily practice leads to improved performance, biomechanical efficiency, and refined tactile control, underpinned by neural and behavioral adaptations (Yamada et al., 2019; Krakauer et al., 2019).

The cognitive action architecture approach (CAA; Schack, 2004, 2020) posits that mental representation structures play a crucial role in motor learning by serving as a foundation for planning and executing motor actions. Consistent with this view, previous research has demonstrated the importance of mental representation structures in developing and controlling voluntary movements (Mechsner et al., 2001; Schack and Mechsner, 2006; Frank et al., 2013; Schack and Frank, 2021). However, the relationship between mental representation structures and motor performance in complex bimanual tasks remains to be elucidated.

Moreover, motor learning research has often overlooked the role of biomechanics and tactile pressure in skill acquisition, despite their potential importance in achieving efficient movement control and optimal performance (Enoka, 1997; Dahiya et al., 2009; Lee-Miller et al., 2019). Understanding the interplay between biomechanics, tactile pressure, and mental representation structure can offer valuable insights into the multifaceted nature of motor learning processes.

In line with these perspectives, we hypothesize that mastery in motor learning is accompanied by a gradual emergence of strong “blending patterns.” This involves a gradual transformation from a “program-like discrete” structure of successive Basic Action Concepts (BACs) (Schack and Frank, 2021) into a more “holistic” interaction pattern, where consecutive BACs are strongly “blended” into each other.

Therefore, we aim to (1) investigate the effects of daily practice on task performance, biomechanics behavior, and tactile pressure, (2) examine the relationship between changes in mental representation structures and changes in skill performance on a complex movement, and (3) explore the interplay between biomechanics, tactile pressure, and mental representation structure in motor learning. By integrating findings from performance, biomechanics, tactile pressure, and mental representation structure analyses, our study seeks to provide a more comprehensive understanding of the underlying processes involved in motor learning and contribute to the broader understanding of motor learning and neurocognition.

Our investigation into the maze game task also affords an excellent opportunity for observing both error-based learnings (Martin et al., 1996) and reinforcement learning (Izawa and Shadmehr, 1996), which for human motor tasks has thus far received little attention. Furthermore, our approach seeks to capture the essence of human learning for novel tasks such as the maze game to gain insights into the process and perhaps use this knowledge to bootstrap machine learning algorithms on our anthropomorphic robot systems (Bentivegna et al., 2004). By studying the fine-grained details of human learning during the acquisition of a complex bimanual skill, we hope to uncover general principles of motor learning that could be transferable to other domains, such as sports training, rehabilitation, and the development of more efficient and human-like robotic systems.

In summary, the present study aims to explore the multifaceted nature of motor learning in a complex bimanual task, examining the interplay between mental representation structures, biomechanics, tactile pressure, and performance (Karniel and Mussa-Ivaldi, 2002; Latash, 2012; Land et al., 2013). By providing a comprehensive understanding of the underlying processes in motor learning, our findings will not only contribute to the growing body of knowledge in the field of motor learning but also have practical implications for designing effective training strategies, interventions, and robotic systems that emulate human motor control and learning capabilities (Peters and Schaal, 2006; Maycock et al., 2010; Sigrist et al., 2013).

## 2 Materials and methods

### 2.1 Participants

Twelve participants from a local university (five female, seven male; *M*_*age*_ = 21.38 years, *SD* = 1.92 years) took part in the present study. All of the participants were initially naive about the purpose of the experiment and gave their informed consent prior to the experiment. They were required to self-report that they were healthy, had normal or corrected to normal visual acuity, and had no known cognitive or neurological problems. Participants self-reported being right-handed; however, no formal handedness inventories were applied. The experiment was conducted in accordance with the ethical standards stated in the 1964 Declaration of Helsinki and revised in 2013, and approved by the Ethics Review Board (EUB) of Bielefeld University.

### 2.2 Apparatus and maze design

**Figure 2:**
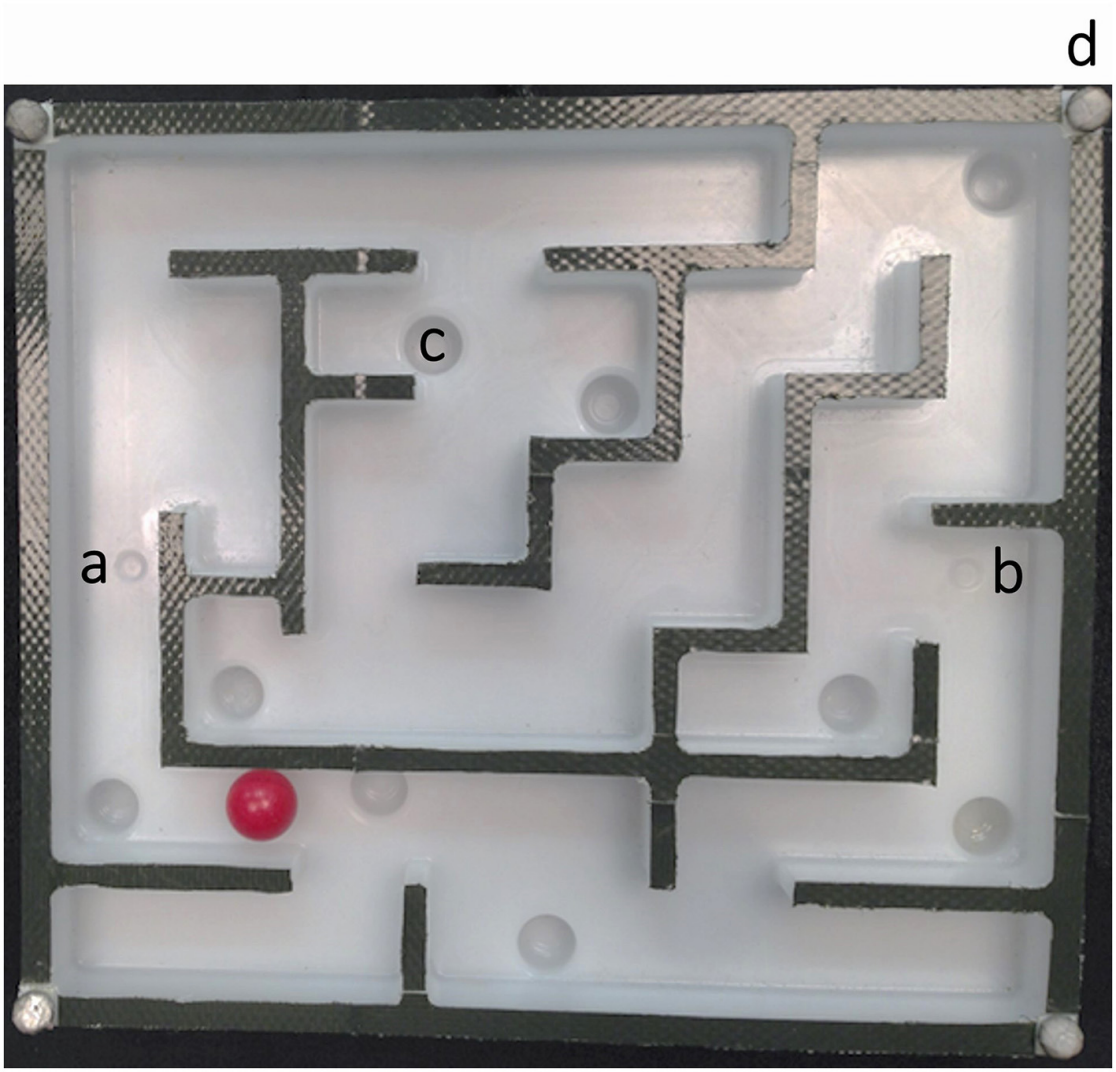
The hardware maze setup. The starting position is marked with an a; the b indicates the goal position. There are a total of nine pits, see example at c, distributed along the maze pathways, and four small reflective markers were placed in the corners of the maze as exemplified at d. A small bright red steel sphere was used to navigate the maze.

The experiment was carried out in the Manual Intelligence Laboratory (MILAB) (Maycock et al., 2010) which houses 14 Vicon (VICON, 2015) MX3+ cameras (200 Hz) for motion capture. The maze was designed with attached tactile sensors (Schürmann et al., 2011) to capture the pressures applied by participants during their execution. The maze measures 17 cm × 15 cm, with additional tactile sensor pads on the left and right, each sized 9 cm × 9 cm (sensor area 8 cm × 8 cm, including a 5 mm frame). The overall weight of the maze, including the tactile pads, is 650 g. A contact microphone was added to the underside of the maze in order to detect contact collision events, and four retroreflective markers were placed on the corners of the maze. The experiments were recorded using a high-speed Basler camera capturing at 200 Hz. The maze includes a series of straight, turns, cross-junctions, and terminal sections. There are nine pits distributed along the two possible pathways (upper and lower path) (see Figure 2 for a detailed view).

### 2.3 Experimental procedure

The present study consisted of a pre-test, a skill acquisition phase of three consecutive days of practice, and a post-test (see Table 1) following the same procedure for all participants.

**Table 1:** Experimental procedure design including three test days and post-test.

**Pre-test**	**Skill acquisition phase**	**Post-test**
**Day 1**	**Day 1–Day 3**	**Day 4**
SDA-M + performance rating	Maze practice	SDA-M

SDA-M: structural dimensional analysis of mental representation; performance rating: 10 min of maze play to rate participants' initial performance; maze practice: 20 min of maze play after three warm-up minutes.

#### 2.3.1 Pre-test

First, participants were informed about the overall investigation and were asked to read and sign an informed consent form. Next, before beginning the experiment and having any contact with the maze itself, the initial state of the mental representation structure was evaluated. Expressly, participants were provided with an explanation regarding the splitting procedure process and the significance of the 14 movement situations present in the maze. Then, participants were instructed to decide whether the situations or basic action concepts were related to one another or not to execute the required movement when solving the maze. Following this, the participants completed the splitting procedure to determine their starting mental representation structure. After that, participants had an initial 5-min period to familiarize themselves with the maze. During this time, the goal was to navigate the sphere through the maze from the starting to the target position while avoiding the pits. Subsequently, to assess participants' initial performance rating, each participant navigated the maze for an extra 10 min. This time, they were told to avoid contact with the walls altogether. A complete trial was considered every time the sphere reached the target position; anytime the sphere fell into a pit, the trial was deemed terminated, and they had to restart from the starting point. We also controlled the amount of time the participants performed in the upper path and the lower path for parity. It was made clear to participants that they could take as long as they needed to complete each trial.

#### 2.3.2 Skill acquisition phase

Participants performed a warm-up playtime of 3 min with the maze, followed by a practice period consisting of 20 min of maze play per day for three consecutive days, following the same rules as before. Participants should avoid contacting the walls or falling into the pits, otherwise, the trial is restarted, and they had no time limit restrictions for individual trials. In detail, participants played for 10 min in the upper path and 10 in the lower path, and the order of play was respectively counterbalanced. No feedback was given during this phase. The only feedback received by the participant was that of the visible outcome (i.e., trial terminated).

#### 2.3.3 Post-test

The post-test was done the day after the end of the skill acquisition phase. The same experimental procedure for assessing mental representation was followed. The goal of the procedure was to determine alterations in participants' mental representation structure induced by mastering the maze.

### 2.4 Measurements

#### 2.4.1 Cognitive primitives in maze task

Previous studies (Bentivegna and Atkeson, 2001; Bentivegna et al., 2004) have proposed a set of primitives extracted from observing humans playing a maze game followed by self practice. Bentivegna and colleagues argued that if primitives are not used to learning, generalization is not feasible. Primitives provide a way to allow the reuse of learned actions. The primitives they identified include “Guide,” “Roll To Corner,” “Roll From Wall,” and “In Corner.” These primitives represented sequences of task states, including the position and velocity of the marble, as well as the maze tilt angles. While their results are remarkable, and they have succeeded in endowing robots with the ability to play a maze game (Bentivegna et al., 2004), we observed that these primitives were derived solely from successful trials and did not account for scenarios involving mistakes or variations in sensorimotor control.

#### 2.4.2 Introduction of cognitive primitives

To address this limitation and provide a more comprehensive representation of the maze game, we introduced the concept of “cognitive primitives.” Cognitive primitives are fundamental units of cognitive and motor actions that represent specific movement patterns and strategies in the context of the maze task. This approach aligns with the concept of action primitives as elementary building blocks for action representation, widely supported in the literature (Handzel and Flash, 1999; Bennequin et al., 2009; Giszter, 2015).

We expanded the original set of primitives to include additional primitives that capture a wider range of movements and scenarios. The newly introduced cognitive primitives include “Random Bounce,” “Controlled Bounce,” “Steady,” “Roll Along Wall,” “Roll To Wall,” and “At Rest,” (see Table 2).

**Table 2:** Definitions of cognitive primitives in maze task used in the study.

Random bounce	The ball bounces around randomly with little or no apparent control
Roll along wall	The ball rolls along the wall continuously
Controlled bounce	The ball bounces off a wall once before exiting a particular section of the maze
Roll to wall	The ball rolls to a wall and then rolls along the wall
Steady	A variant to the Guide primitive, the ball rolls forward without touching any walls and then returns to a similar position
At rest	The ball is motionless for a defined period of time

**Figure 3:**
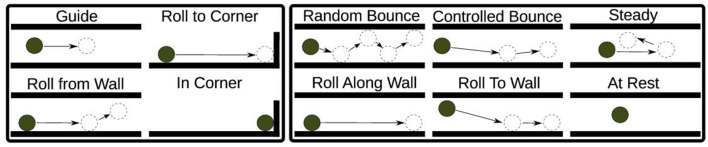
The chosen set of maze primitives. The left-hand side box contains primitives from Bentivegna et al. (2004) in the tilt maze environment, which we extended to include those shown in the box on the right.

These cognitive primitives encompass a broader spectrum of actions, including bouncing, rolling along walls, resting, and controlled maneuvers, thereby accommodating scenarios involving mistakes or imperfect control (see Figure 3).

#### 2.4.3 Cognitive structures and mental representation

The term “cognitive primitives” allows us to link the observed behavior to the practical strategies employed by participants to solve the maze task. These primitives are not merely kinematic patterns but we see them as cognitive and functional units representing the mental representation structures underlying the planning and execution of movements. To provide a clearer understanding of the concept, cognitive primitives refer to the basic units of thought and action planning required for efficient navigation of the maze. They are crucial for constructing and refining the mental representations that guide movement, integrating sensory feedback and motor commands to optimize performance. Consistent with this view, previous research has demonstrated the importance of mental representation structures in developing and controlling voluntary movements (Mechsner et al., 2001; Schack and Mechsner, 2006; Frank et al., 2013; Schack and Frank, 2021). This concept is supported by recent research on the role of the cerebellum in constructing functional and geometrical spaces, which emphasizes the importance of motor primitives in explaining movement and perception-action coupling (Langlois et al., 2024).

#### 2.4.4 Skill performance

After deciding on the cognitive primitives that best suit our maze game, we follow a similar approach as laid out by Bentivegna et al. (2003) to recognize these primitives and annotate and segment trials automatically. To avoid subjective variations caused by manual annotation processes, we automated the primitive detection procedure. Four Vicon markers were placed on the maze's corners, and the Basler camera was calibrated with the Vicon setup. The steel sphere was painted bright red to facilitate tracking. Furthermore, a model of the maze, divided into 21 sections to assist with tracking and improve the identification of the cognitive primitives, was then overlaid on top of the maze image. Each of these 21 sections was further analyzed by the computer vision algorithm.

**Figure 4:**
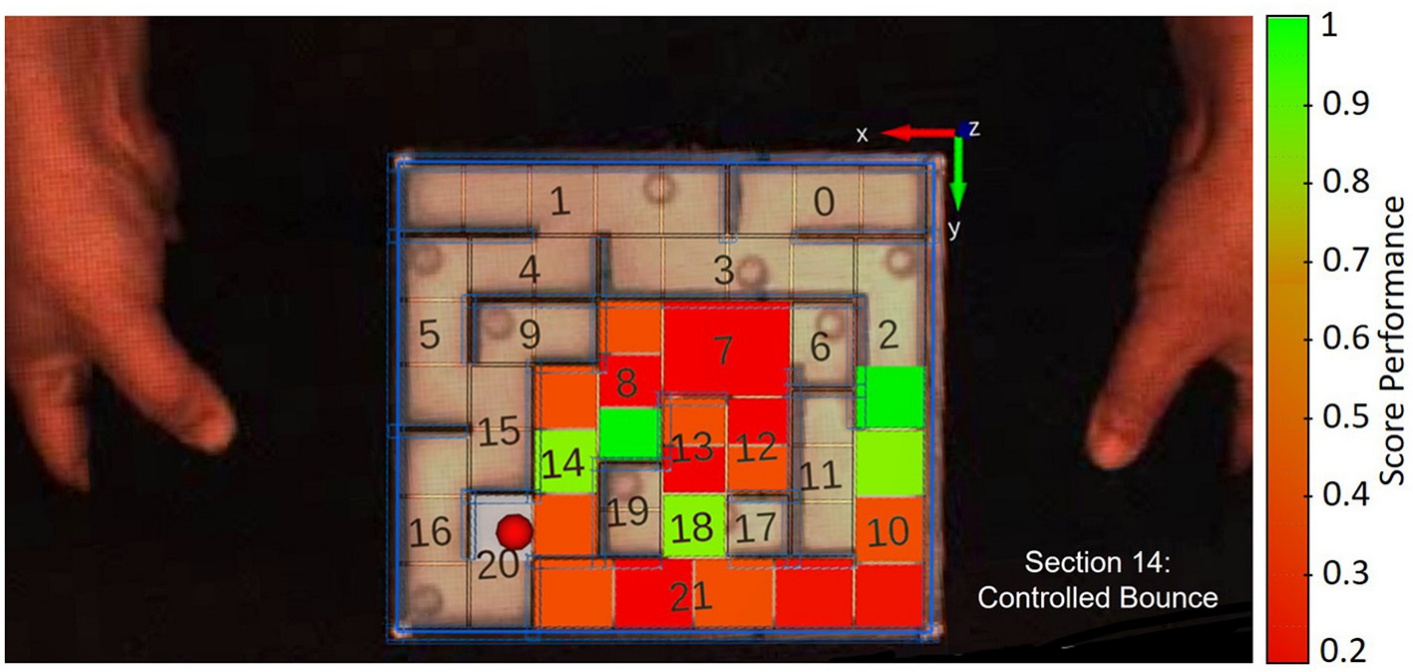
A single trial example, highlighting the process of scoring and evaluating trial performance in the maze game. The maze is divided into 21 sections, and each section is further analyzed by the computer vision algorithm. Penalties are assigned for wall contact, and the algorithm reports the latest identified primitive and its corresponding section. Additionally, the algorithm visually represents performance by coloring each section, ranging from red to green. Full green represents an ideal scenario with “Guide” primitives, while full red indicates a chain of penalized primitives, indicating poor performance.

Breaking each trial into a sequence of cognitive primitives allowed us to compute a performance score. This was done by assigning a penalty to primitives which contained a contact with the wall. Thus, a symbolic representation of each trial was produced, and an overall fitness score was computed by calculating the distance each trial was from an ideal trial containing only “Guide” primitives (i.e., completely avoiding all contact with the walls). An ideal trial, composed solely of “Guide” primitives and other non-wall-contact primitives, was assigned a score of 1. Therefore, 1 is the maximum score, and every time wall-contacting primitive occurs, a corresponding penalty was subtracted from this ideal score until the lowest possible score of 0. The length of the strings (i.e., the time duration of each trial) was not penalized. See Figure 4 for a single trial example.

#### 2.4.5 Biomechanic metrics

The aforementioned 14-camera Vicon system was used to capture the position of 4 retroreflective markers attached to the maze corners at 200 Hz. The raw three-dimensional coordinates were exported and preprocessed. Specifically, the data was filtered through a fourth-order low-pass Butterworth digital filter with an estimated optimum cutoff frequency ranging from 6 to 17 Hz, depending on the data frequency and value ranges (Yu et al., 1999). This filtering process helps mitigate the influence of high-frequency noise that could skew the results.

For each trial, we extracted velocity profiles using a custom MATLAB R2021a program (MATLAB, 2021) calculating the velocity in three-dimensional space to obtain the magnitude of the joint velocity in Euclidean terms for each marker (Robertson et al., 2013). These profiles were derived by calculating the average rate of change of position of all markers with respect to time. The profiles of all markers were then averaged to produce a single, comprehensive velocity profile of the maze. From these velocity profiles, we identified two key metrics: the peak velocity and the number of velocity peaks. The peak velocity is defined as the maximum instantaneous velocity achieved during the trial. To ensure robustness against outliers, we applied an additional step of detecting and removing spurious spikes that deviate significantly from the local velocity trend before determining the peak velocity. The number of velocity peaks is determined by counting the instances of local velocity maxima that exceed 40% of the peak velocity, with each peak separated by a minimum interval of 100ms to ensure distinctness (Thomas et al., 2022). These peaks generally represent the sub-movements within the task. These sub-movements refer to different motions that are present in the primitives; they tend to exhibit these sub-movements, characterized by a pattern of initiating and then halting motion. The task, therefore, consists of a sequence of sub-movements, each displaying a bell-shaped velocity profile. While this metric counts the number of peaks, it does not account for the amplitude of velocity variations. Therefore, it should be noted that a high peak velocity does not necessarily equate to effective or controlled movement performance. Small and large velocity variations are treated equally in this analysis. However, these sub-movement peaks are essential in understanding the task's completion as they highlight the participant's movement strategy and control.

#### 2.4.6 Tactile pressure

Using the tactile sensors, we obtained the pressure values for the thumb finger of the left and right hand, respectively. The pressure range of the sensor was calibrated for 3–100 kPa, and the response values range from 0 to 4,095. However, due to the sensor characteristics, the response values cannot be taken as a simple linear measure of the net normal force applied to the sensor. A 16 × 16 tactile image was extracted from the values and preprocessed with a 3-by-3 neighborhood median filter to remove any possible undesired noise and artifacts. Thereafter, a mean tactile pressure image was produced for each trial using a custom program written in MATLAB R2021a (MathWorks, Inc., Massachusetts, USA) by averaging the tactile values.

We used the 16 × 16 tactile image to represent the spatial distribution of pressure across the sensor's surface, as the raw sensor response values cannot be linearly interpreted as a direct measure of the net normal force applied. This approach allowed us to better understand the pressure interaction between the thumb finger and the maze, as well as identify any variations or patterns in the contact pressure during the trials. Thus, using tactile images provided a more comprehensive and informative representation of the pressure involved in the experiment, enhancing the overall analysis.

To address potential non-linearity and ensure consistency across sessions, we normalized the pressure images by averaging the values relative to each session. This normalization accounted for any deviations in measurements between sessions, given the inherent characteristics of the sensors and the possibility of calibration differences.

#### 2.4.7 Mental representation structure

The structural dimensional analysis of mental motor representations (SDA-M) was utilized to evaluate the participants' mental representation structures for the maze game. This method provides a psychometric assessment of the relational structures and dimensions of mental representations of complex movements stored in long-term memory (see Schack, 2012, 2020 for more details). Instead of relying on explicit participant statements, the SDA-M reveals representational structures through knowledge-based decisions in an experimental context. In essence, SDA-M identifies relationships between basic action concepts (BACs) of a motor action in memory.

The SDA-M involves four steps: First, a splitting procedure is used to obtain distance scaling, resulting in a Euclidean distance that measures the proximity between representational object units (BACs) associated with a specific motor action in long-term memory (LTM). Second, hierarchical cluster analysis organizes the BACs into a hierarchical structure, forming a dendrogram. Third, factor analysis combined with a cluster-oriented rotation process is performed to dimension the cluster solutions, producing a factor matrix categorized by clusters. Finally, an invariance analysis compares cluster solutions both between and within individuals. For a more detailed explanation, refer to Schack (2012, 2020).

**Figure 5:**
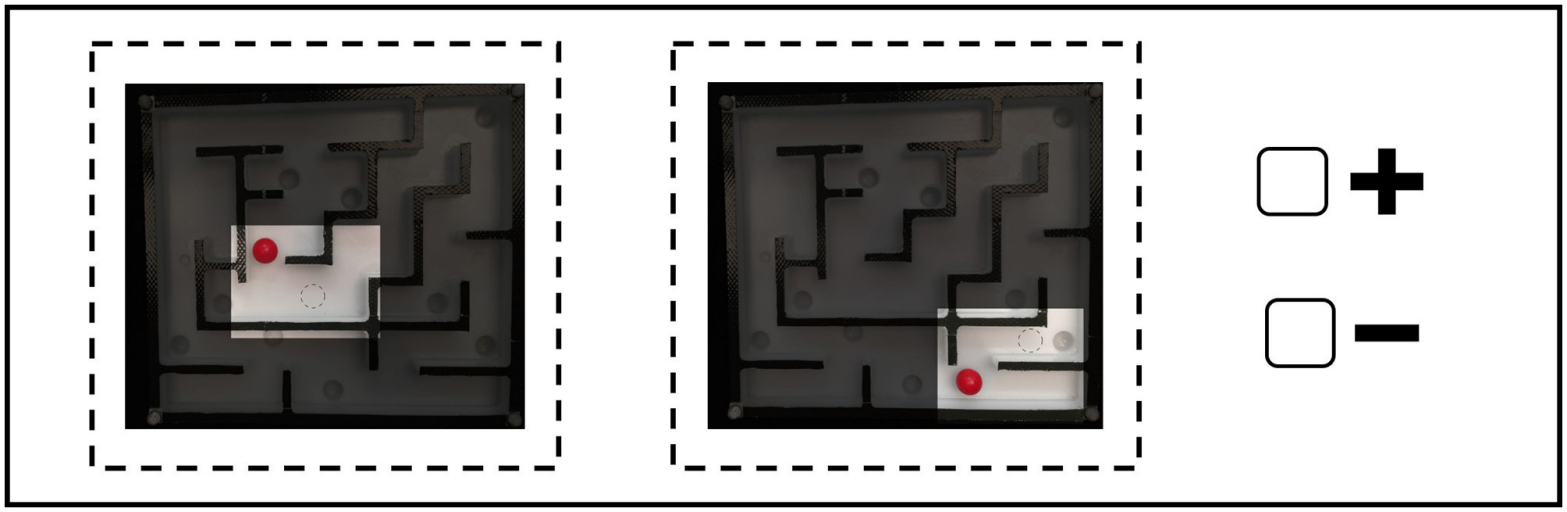
An example of an item comparison from the SDA-M questionnaire for the maze game. Participants were required to choose either the negative or positive sign based on whether the two presented basic action concepts (BACs) at each time, which refer to the type of movement within the given maze situation, are related to each other during motor performance or not.

**Figure 6:**
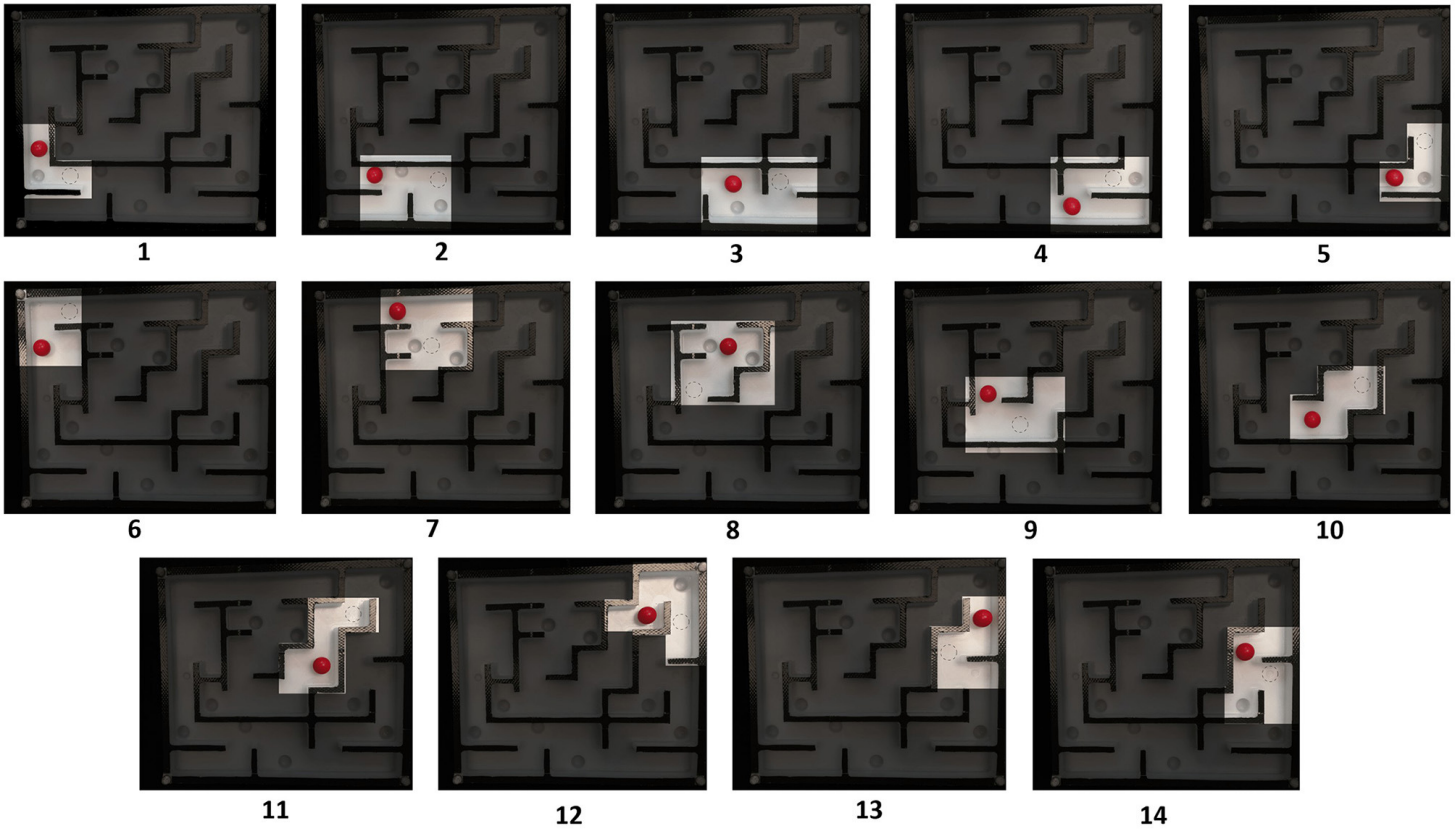
Sequence of 14 pictures depicting the functional movement situations in the maze game, each corresponding to one of the points from 1 to 14. Participants were required to judge these situations, with each picture representing a specific type of movement within the maze game (refer to Table 3).

Following the SDA-M methodology, the first step, the splitting task, was performed on the representational distance between the selected BACs. The participants were asked to subjectively judge the functional equivalence of pairs of BACs (BAC × BAC: pairs of BACs are judged as “functionally related” or “not functionally related” to each other) responding on a positive/negative basis (i.e., positive if related, negative if different) on a computer-based experiment. *Functionally related* refers to the mobilization of body segments, muscles, and proprioception within an egocentric reference frame, according to their own motor execution of the movement. Specifically, participants were required to judge whether two movement maze situations are related to one another or not regarding the movement execution to solve the maze sequence (see Figure 5). For the specific purpose of the present study, a pre-determined set of 14 BACs of the maze game were used (see Table 3), each BAC pertaining to one particular movement type of the maze game: down-right (BAC 1, 9, 14), right-around (BAC 2–3), down-left (BAC 7–8, 13), up-right (4, 6, 11), and right-diagonal-up (BAC 5, 10, 12). Among the fourteen BACs chosen for this study, a randomly selected BAC is presented as an anchor on a computer screen; the remaining thirteen BACs are presented one after another in a randomized order (see Figure 6 for an overview of the fourteen BACs). The same procedure is repeated, randomly selecting a different anchor BAC until every BAC has been compared to every other. Thus, in total, participants were asked to make a total of 182 judgments (14 anchors × 13 comparisons).

**Table 3:** Each of the 14 basic action concepts (BACs) mapping the movement situations of the maze game can be functionally assigned to one of the movement types.

**No**.	**Movement (BAC)**	**Movement type**
(1)	Down-right	Down-right
(9)	Down-right	
(14)	Down-right	
(2)	Right-around-right	Right-around
(3)	Right-around	
(7)	Right-down	Down-left
(8)	Down-left	
(13)	Down-left	
(4)	Up-right	Up-right
(6)	Up-right	
(11)	Up-right	
(5)	Right-diagonal-up	Right-diagonal-up
(10)	Right-diagonal-up	
(12)	Right-up-around	

The numbers on the left relate to the different BACs; they do not reflect any particular order but serve to better display the concepts in Figures 10, 11.

### 2.5 Data analysis

Before proceeding with any analysis, the participants' data were split into two groups according to their initial maze performance (first 5 min of practice during the first day). These maze performance scores were based on the overall fitness score described prevously, where higher scores indicate better performance with fewer penalty primitives for wall contact. Individual scores from these initial minutes were collected and analyzed using a linear regression method to identify performance trends and progression throughout the experiment. Based on this regression analysis, participants were categorized into two groups: poor performance group (*n* = 6, three female, three male; mean age = 21.83 years, SD = 1.32 years), and good performance group (*n* = 6, two female, four male; mean age = 20.73 years, SD = 1.83 years), ensuring equal group sizes. After establishing these two groups, we continued with the following analyses.

#### 2.5.1 Maze score performance and biomechanics

We evaluated the possible relation between the maze score performance and the maze's biomechanics measurements using a Multivariate Analyses of Variance (MANOVA) testing for interaction and main effects by day (repeated measures). The significance level for data analysis was set at 5%. The sphericity assumption was also assessed using Mauchly's test in the between-subject repeated measure analysis. Whenever the test was violated, necessary technical corrections were performed using the Greenhouse–Geisser test (Greenhouse and Geisser, 1959; Abdi, 2010).

#### 2.5.2 Tactile pressure

For this experiment, we were not interested in the absolute forces exerted by the thumbs but in the pressure that each thumb applies on the sensors during the experiment and how this evolves as the training days progress. To this purpose, we generated a mean tactile pressure two-dimensional map image for each training day between the groups. From these generated images, we computed the image centroids (center of pressure) using the method of moments first described by Hu (1962), and using the idea of contact centroid introduced by Bicchi et al. (1993) and later adapted by Cannata et al. (2008) for each tactile image. The centroids represent the high-pressure region and approximate the shape of the pressure distribution to an ellipsoid.

Following a similar approach as with the performance and biomechanics analysis, we evaluated the ellipsoid areas using Multivariate Analyses of Variance (MANOVA) testing for interaction and main effects by day (repeated measures) for each thumb's hand respectively. We set the significance level at 5%, kept the same assumptions for the between-subject analysis, and performed the technical corrections using the Greenhouse–Geisser test when necessary.

#### 2.5.3 Mental representation structure

A cluster analysis of the mean dendrogram for each group was performed. The purpose of the cluster analysis is to reveal the significant clusters in the mean dendrogram by the group; for details, see Schack (2012, 2020). For all cluster analyses conducted, the significance level was set to a 5% (α = 0.05), which corresponds to a 95% confidence level for decisions made (i.e., *p* < 0.05). The threshold value of 3.42 (*d*_*Crit*_ = 3.42) is a critical value for the dissimilarity measure used in the clustering algorithm. This threshold was determined based on the statistical method employed to ensure that the identified clusters are statistically significant. All the clusters in the dendrograms below 3.42 are considered statistically significant, whereas those above this value are taken as statistically irrelevant. Then, an invariance analysis was performed to investigate the statistical differences in the mental representation structures between the groups. The invariance (λ) is a measure of the similarity between cluster solutions, where lower values indicate greater differences. According to Lander (1991) and Lander and Lange (1992); see also Schack (2012), two cluster solutions are variant, that is significantly different, for λ < 0.68, while two cluster solutions are invariant for λ ≥ 0.68. This threshold value of 0.68 was also used in our study to determine the invariance or variance between the cluster solutions. It corresponds to a critical point derived from empirical studies and statistical methods, ensuring that clusters with λ values below this threshold are significantly different. In addition, the adjusted rand index (ARI; Rand, 1971; Santos and Embrechts, 2009) was used to examine the degree of similarity between the group's mental representation structure and the reference structure. The reference structure, which represents the idealized cognitive structure that expert participants are expected to develop through extensive practice and experience with the task, has been manually designed for our particular maze to the best of our expertise (see Supplementary Figure S1). To learn more about how reference structures are built, please see Schack and Mechsner (2006) and Frank et al. (2013). The ARI serves as an index of similarity on a scale from –1 to +1. On this scale, values close to –1 indicate that the two compared cluster solutions are different, with –1 indicating a “completely different” degree of similarity. The values close to 1 indicate that the two compared cluster solutions are similar, with 1 indicating that the cluster solutions are “completely same” or identical.

## 3 Results

### 3.1 Skill level and days of practice impact

A 2 (skill level group) × 3 (practice days) MANOVA, with repeated measures, was computed. The MANOVA was conducted for the skill performance and the biomechanic metrics (maximum velocity and number of peaks). The skill level group acted as the independent variable over all the skill acquisition phase (3 days of practice) for the analysis. Interactions effects were observed for the practice days, and the between-group factor (*p* < 0.05) suggesting that any change-over-time patterns were not similar across the groups. Subsequently, data is presented across the two skill level groups for the three training days (Table 4).

**Table 4:** Comparative table showing the mean area and standard deviations for the three variables and the two groups across the 3 days.

**Days**	**Maze score**		**Maximum velocity (mm/s)**		**Number of velocity peaks**	
	**Poor (PPG)**	**Good (GPG)**	**Poor (PPG)**	**Good (GPG)**	**Poor (PPG)**	**Good (GPG)**
Day 1	0.58 ± 0.0	0.65 ± 0.0	69.64 ± 1.9	40.69 ± 2.1	7.87 ± 0.3	8.43 ± 0.3
Day 2	0.61 ± 0.0	0.68 ± 0.0	69.83 ± 1.9	26.70 ± 2.1	8.08 ± 0.2	6.72 ± 0.3
Day 3	0.68 ± 0.0	0.81 ± 0.0	67.95 ± 2.4	26.00 ± 2.6	6.98 ± 0.1	5.94 ± 0.2

A significant multivariate (skill group × days) interaction were observed in the MANOVAs testing, Pillai's trace = 0.111, *F*_(6, 384)_ = 7.99, *p* < 0.001, $\eta_{p}^{2}$ = 0.111. In detail, significant between-group (PPG × GPG) differences were revealed, Pillai's trace = 0.483, *F*_(3, 387)_ = 120.35, *p* < 0.001, $\eta_{p}^{2}$ = 0.483 regardless of time period. Furthermore, across all analysys, significant variances were noted for within-subject (practice days), Pillai's trace = 0.483, *F*_(6, 384)_ = 49.87, *p* < 0.001, $\eta_{p}^{2}$ = 0.483.

### 3.2 Maze score performance

**Figure 7:**
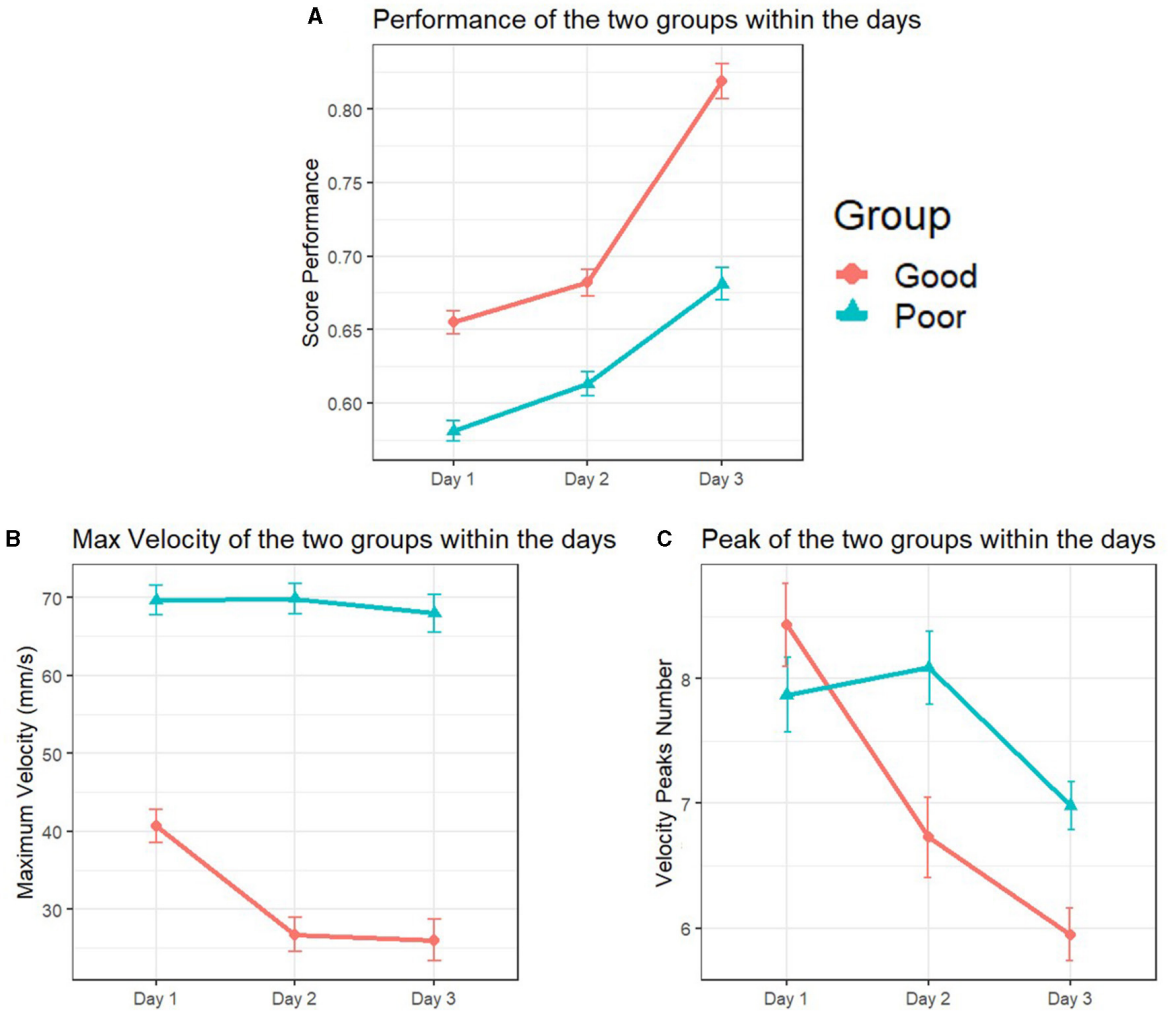
Maze performance, maximum velocity, and velocity peaks differences across skill groups and practice days. In **(A)**, the Poor Performers' Group (PPG) consistently obtained lower scores compared to the Good Performers' Group (GPG) during the skill acquisition phase, with noticeable improvements observed on the third day. **(B)** reveals consistent differences in maximum velocity, with the GPG consistently performing at lower values compared to the PPG throughout all practice days. **(C)** highlights variations in the number of velocity peaks. The GPG exhibited a decreasing trend in the number of peaks executed each day, while the PPG displayed a similar number of peaks on the first and second day but significantly fewer on the third day.

The univariate test revealed a significant between-subjects (skill group × days) interaction effect for the maze performance scores, *F*_(1.83, 713.47)_ = 8.589, *p* < 0.001, $\eta_{p}^{2}$ = 0.22. A further inspection of the follow-up test revealed both inter and intra-group differences. Notably, between-subject ANOVA noting group differences, *F*_(1, 389)_ = 119.77, *p* < 0.001, $\eta_{p}^{2}$ = 0.23, and within-subject ANOVA noting significant changes throughout all the practice day phase, *F*_(1.834, 713.47)_ = 120.89, *p* < 0.001, $\eta_{p}^{2}$ = 0.23. Specifically, the PPG exhibited lower scores than the GPG throughout all the skill acquisition phases (Table 4). In addition, corrected *t*-tests further showed performance improvement within the days, with a more pronounced development during the third day for the two groups (Figure 7A).

### 3.3 Maze maximum velocity

A significant between-subjects (skill group × days) interaction effect was observed for the maximum velocity applied in the maze, *F*_(1.94, 757.42)_ = 7.70 *p* < 0.001, $\eta_{p}^{2}$ = 0.01. A further inspection of the follow-up test revealed between differences through the 3 days, *F*_(1, 389)_ = 315.70, *p* < 0.001, $\eta_{p}^{2}$ = 0.44. In addition, a within-subject ANOVA showed variances during the days *F*_(1.947, 757.42)_= 9.65, *p* < 0.001, $\eta_{p}^{2}$ = 0.24. Specifically, the PPG performed higher maximum velocity values when manipulating the maze compared to the GPG throughout all the practice days (Table 4). The corrected *t*-tests further showed that the differences were significant for the GPG group during all stages of the skill acquisition phase. The PPG, however, although showing a slightly similar trend of declining values among the days, the corrected *t*-test revealed the differences to be non-significant (Figure 7B).

### 3.4 Maze number of velocity peaks

A significant between-subjects (skill group × days) interaction effect was observed for the number of velocity peaks applied in the maze, *F*_(1.879, 730.76)_ = 7.04 *p* = 0.001, $\eta_{p}^{2}$ = 0.018. Particularly, a between-group ANOVA exposed differences among the two groups, *F*_(1, 389)_ = 6.57, *p* = 0.11, $\eta_{p}^{2}$ = 0.017. The corrected *t*-test further showed that the differences were present only between the second and third day of practice for the two groups, with both groups exhibiting similar behavior during the first day (Table 4). Additionally, noticeable differences arose from the within-subject ANOVA during the skill acquisition phase, *F*_(1.897, 730.76)_ = 18.99, *p* < 0.001, $\eta_{p}^{2}$ = 0.047. The corrected *t*-tests further showed that the differences were significant for the GPG group over the 3 days, exhibiting a negative trend with fewer peaks executed each day. The PPG performed a similar number of peaks on the first and second day, and a significantly lower number of peaks only during the third day of practice (Figure 7C).

### 3.5 Tactile pressure

A 2 (skill level group) × 3 (practice days) MANOVA, with repeated measures, was computed for the area of the center of pressure (centroids) for the left and the right-hand thumbs. Interaction effects were found for the practice days, between the different hands, and the between-group factor (*p* < 0.05) suggesting that there were significant differences in how pressure patterns developed over time for both groups and for both hands. The mean tactile pressure areas for both hands are presented across the two skill level groups for the three training days (Table 5).

**Table 5:** Comparative table showing the mean centroid area and standard deviations for the right and left thumb and the two groups across the 3 days.

**Days**	**Right thumb**		**Left thumb**	
	**Poor (PPG)**	**Good (GPG)**	**Poor (PPG)**	**Good (GPG)**
Day 1	15.66 ± 0.33	16.49 ± 0.40	17.05 ± 0.70	16.61 ± 0.78
Day 2	13.49 ± 0.44	12.85 ± 0.54	15.03 ± 1.26	15.22 ± 1.40
Day 3	14.45 ± 0.30	10.73 ± 0.36	14.87 ± 0.50	13.70 ± 0.55

#### 3.5.1 Right hand thumb

A significant multivariate (skill group × days) interaction was observed in the MANOVA testing, Pillai's trace = 0.227, *F*_(2, 145)_ = 21.29, *p* < 0.001, $\eta_{p}^{2}$ = 0.227. A further inspection of the follow-up test revealed inter and intra-group variances. Specifically, between-group (PPG × GPG) differences were noted for the group *F*_(1, 146)_ = 12.63, *p* < 0.001, $\eta_{p}^{2}$ = 0.080 regardless of the training period time. In general, the PPG group produced mean larger area sizes (*M* = 14.53 pixels) than the GPG group (*M* = 13.36 pixels). An additional corrected *t*-test showed no significant differences during the first and second day between the two groups, whereas a significant difference was found for the third day of practice, *F*_(1, 146)_ = 61.23, *p* < 0.001, $\eta_{p}^{2}$ = 0.295, with the GPG exhibiting a significantly smaller area size than the PPG group (see Table 5).

**Figure 8:**
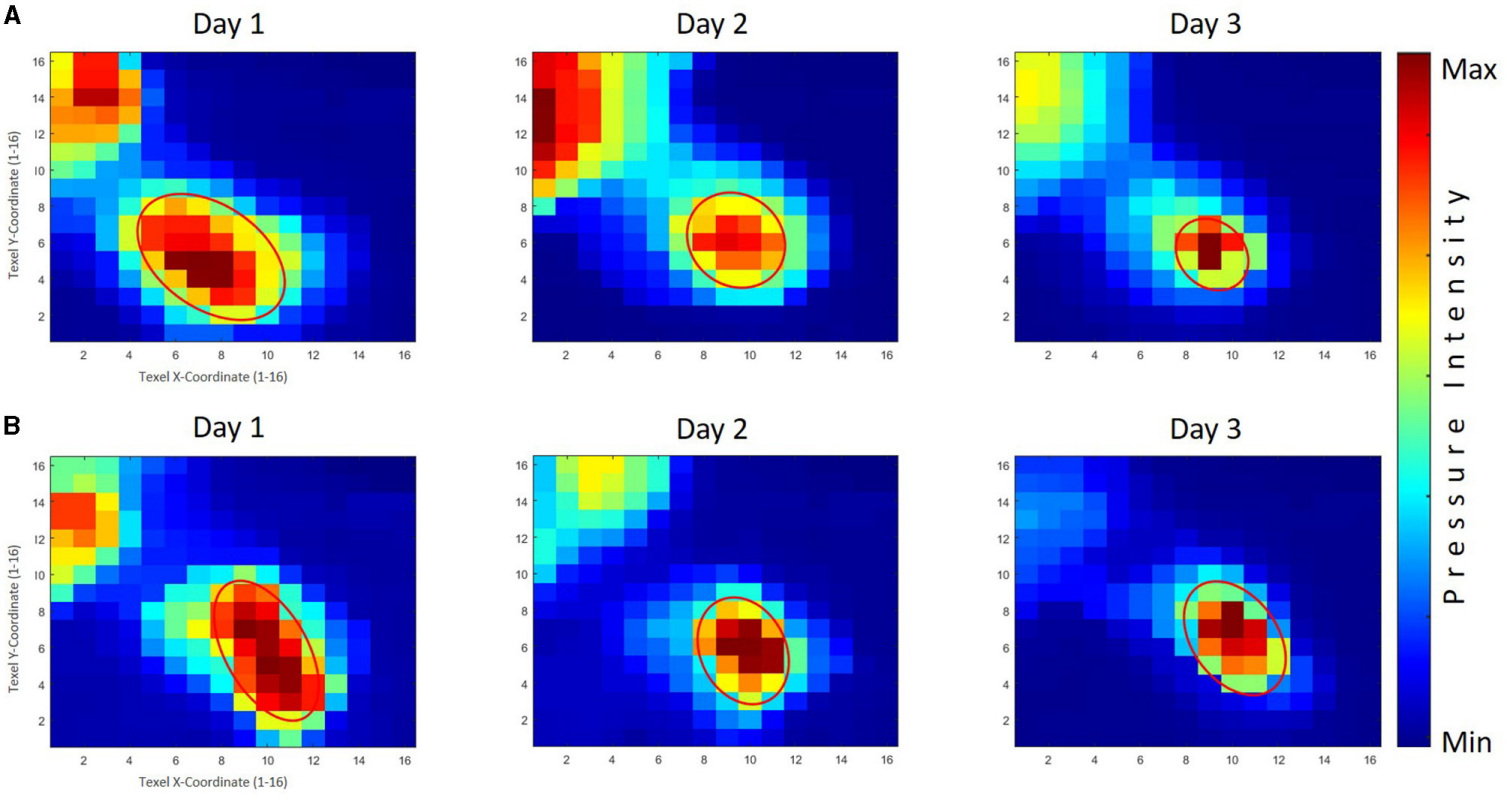
The averaged tactile pressure distribution on the right thumb for the good performers' group (GPG) and poor performers' group (PPG). Row **(A)** displays the results of the GPG across three practice days, while Row **(B)** shows the corresponding results for the PPG. The GPG exhibited significant decreases throughout the skill acquisition phase, with a significantly smaller area size than the PPG observed on the third day. The PPG demonstrated significant reductions in area values on the first and second days, with generally larger area sizes compared to the GPG.

Furthermore, across all analyses, significant variances were noted for within-subject (practice days), Pillai's trace = 0.575, *F*_(2, 145)_ = 53.49, *p* < 0.001, $\eta_{p}^{2}$ = 0.425 for the area sizes, with a follow-up within-subject ANOVA noting considerable changes for the PPG, Pillai's trace = 0.106, *F*_(2, 145)_ = 8.58, *p* < 0.001, $\eta_{p}^{2}$ = 0.106, and for the GPG, Pillai's trace = 0.438, *F*_(2, 145)_ = 56.49, *p* < 0.001, $\eta_{p}^{2}$ = 0.438. An inspection of the corrected *t*-tests further indicated the area values reduced significantly within the first and second day of practice, *p* < 0.001, but not within the second and third day for the PPG, whereas the area values significantly decreased within all stages of the skill acquisition phase, *p* < 0.01, for the PPG. The averaged tactile pressure distribution is shown in Figure 8 corresponding to the thumb of the right hand of the participants.

#### 3.5.2 Left hand thumb

A significant main effect (practice days) was observed in the MANOVA testing, Pillai's trace = 0.120, *F*_(2, 111)_ = 7.55, *p* < 0.001, $\eta_{p}^{2}$ = 0.120. A further inspection of the follow-up test revealed no between-group (PPG × GPG) significant differences, indicating that both groups performed similarly, with the PPG producing a slightly mean larger area sizes (*M* = 15.65 pixels) than the GPG group (*M* = 15.18 pixels), and without any significant differences during the practice days between the two groups (see Table 5).

**Figure 9:**
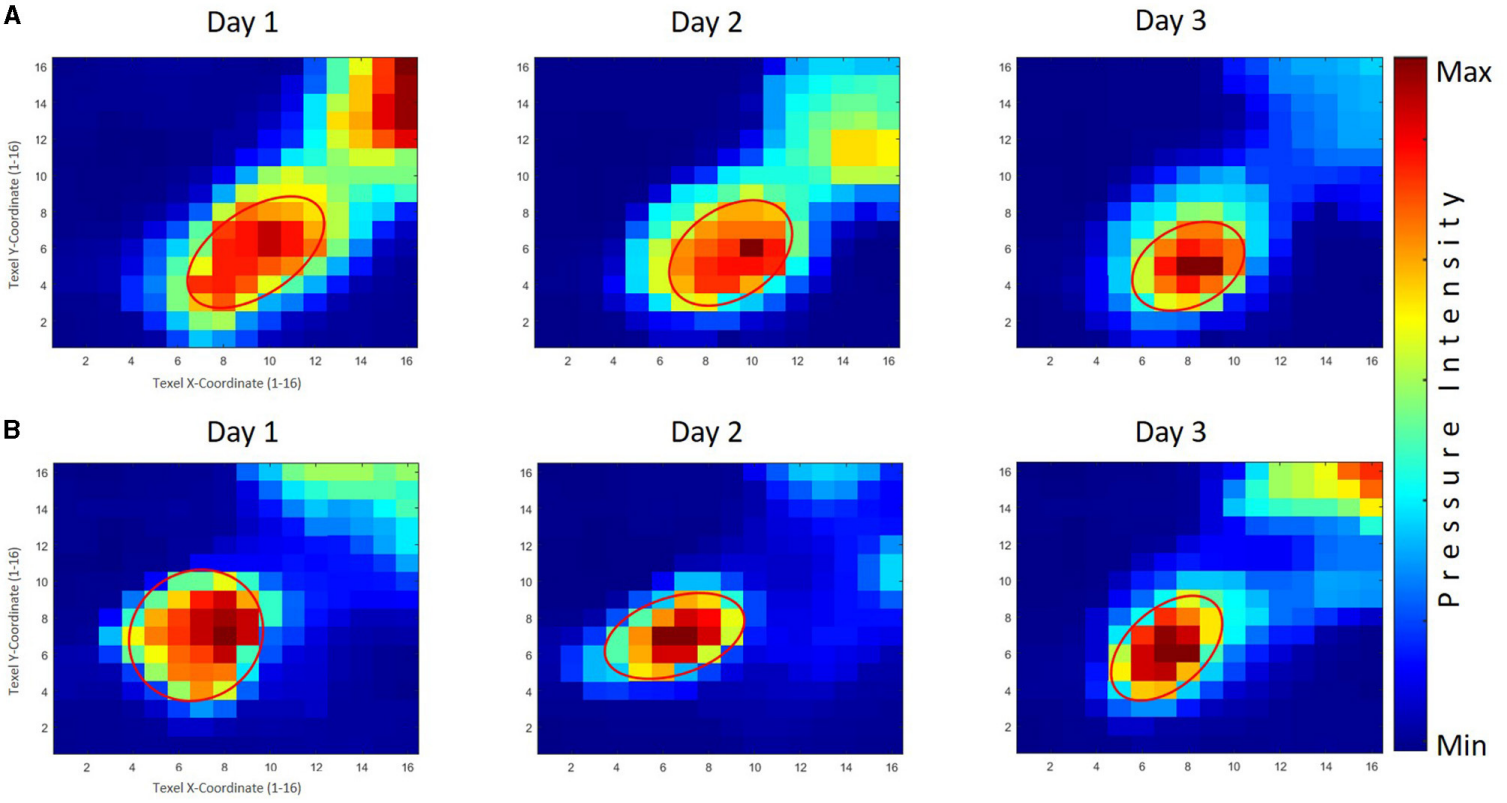
The averaged tactile pressure distribution on the left thumb for both the good performers' group (GPG) and poor performers' group (PPG) across three practice days. Row **(A)** displays the results of the GPG, while Row **(B)** shows the corresponding results for the PPG. Both groups displayed a decreasing trend in area size values across practice days, with a significant difference observed between the first and third days. The PPG exhibited slightly larger area sizes compared to the GPG, although no significant differences were found between the groups throughout the practice days.

Furthermore, significant variances were noted for within-subject (practice days), Pillai's trace = 0.120, *F*_(2, 111)_ = 7.55, *p* < 0.001, $\eta_{p}^{2}$ = 0.120 for the area sizes, with a follow-up within-subject ANOVA noting considerable changes for the PPG, Pillai's trace = 0.057, *F*_(2, 111)_ = 3.37, *p* < 0.05, $\eta_{p}^{2}$ = 0.057, and for the GPG, Pillai's trace = 0.073, *F*_(2, 111)_ = 4.33, *p* < 0.05, $\eta_{p}^{2}$ = 0.073. An inspection of the corrected *t*-tests further indicated the area values reduced significantly only within the first and third day of practice, *p* < 0.05 for the PPG. A further inspection within the GPG showed a reducing trend in the area sizes with a significant difference, *p* < 0.05, between the first and the third day. The averaged tactile pressure distribution is shown in Figure 9 corresponds to the thumb of the left hand of the participants.

### 3.6 Mental representation structure

**Figure 10:**
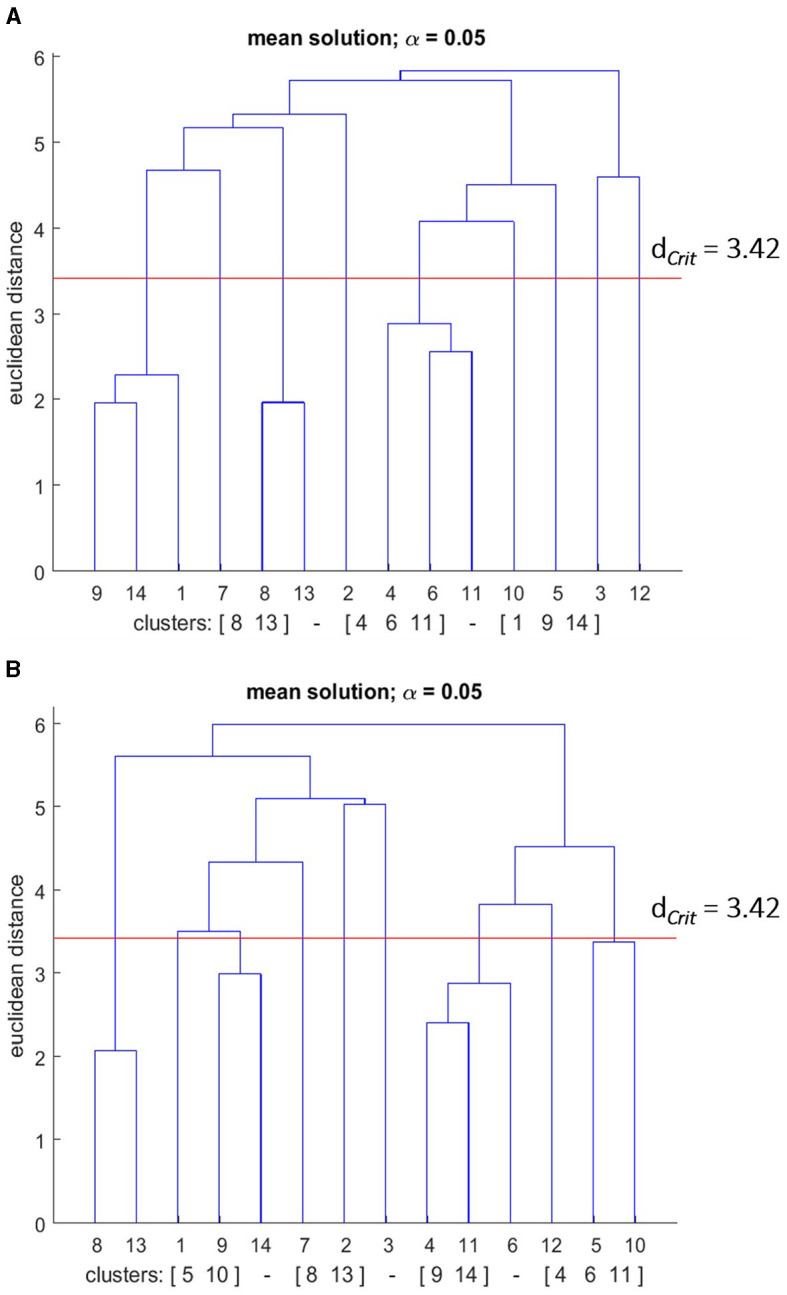
Mean dendrograms indicate the mental representation structure of the poor performance group (PPG) at **(A)** pre-test and **(B)** post-test. The horizontal line indicates the critical Euclidean distance. The critical value of the Euclidean distance (*d*_*Crit*_ = was 3.42 for an α level of 5%). The basic action concepts (BACs) above this line are considered unrelated. The underlined BACs below this line are considered functionally related to each other.

**Figure 11:**
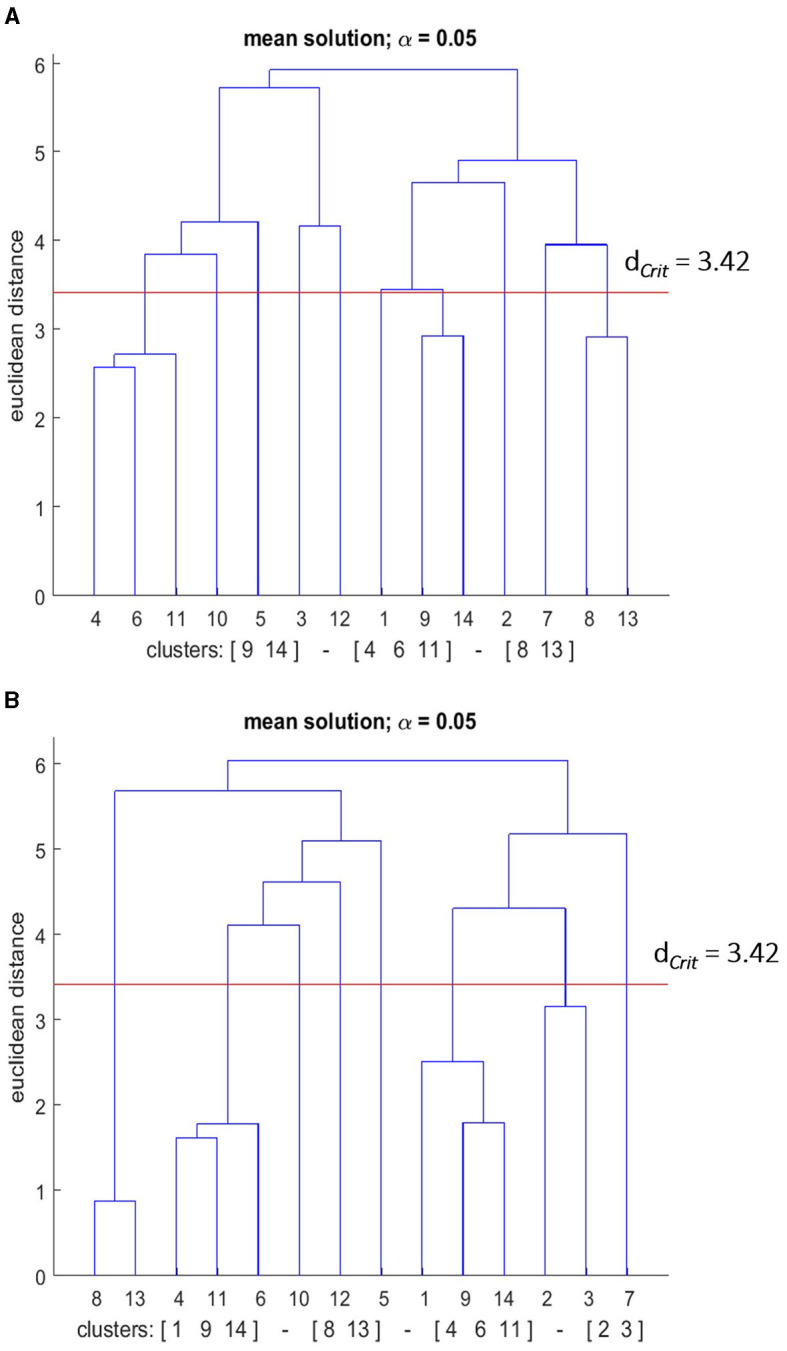
Mean dendrograms indicate the mental representation structure of the good performance group (GPG) at **(A)** pre-test and **(B)** post-test. The horizontal line indicates the critical Euclidean distance. The critical value of the Euclidean distance (*d*_*Crit*_ = was 3.42 for an α level of 5%). The basic action concepts (BACs) above this line are considered unrelated. The underlined BACs below this line are considered functionally related to each other.

As a result of cluster analysis, statistically, significant clusters were found in the mean group dendrograms for the pre and post-test (see Figures 10, 11).

#### 3.6.1 Poor performers group

The cluster analysis showed that the number of statistically significant functional clusters had increased over the skill acquisition sessions (see Figure 10). More specifically, the clusters were (BAC 1, 9, 14), (BAC 4, 6, 11), (BAC 8, 13) at the pre-test, and (BAC 9, 14), (BAC 4, 6, 11), (BAC 8, 13), and (BAC 5, 10) at the post-test. Thus, for the PPG group, an increase in the number of clusters was revealed in their mental representation structure over the course of the skill acquisition phase. The statistical analysis of invariance, however, showed that this increment does not represent a significant difference between the pre, and post-test structures (λ > 0.69). When examining the adjusted rand indices of the pre-test (ARI_pre_ = 0.59) and the post-test (ARI_post_ = 0.66), the results indicated that over the course of the practice days, given that the ARI value ranges from –1 (i.e., completely different) to +1 (i.e., completely same), the mean dendrogram of the PPG group became more similar to the reference dendrogram. That indicates that the changes in the representation structure of the PPG group reflect a development toward an optimal structure; nevertheless, this slight improvement is considered not statistically relevant according to the invariance analysis.

#### 3.6.2 Good performers group

Similar to the PPG group, the mental representation structure of the GPG group was more elaborated after the skill acquisition phase (see Figure 11). In detail, three clusters were evident in the combined GPG group's mean dendrogram at the pre-test: (BAC 9, 14), (BAC 4, 6, 11), and (BAC 8, 13). For the post-test, the mean group dendrogram revealed the fourth cluster, with the previous rest remaining nearly the same. More explicitly, the clusters were (BAC 1, 9, 14), (BAC 4, 6, 11), (BAC 8, 13), and (BAC 2, 3). Hence, for the GPG group, the number of clusters increased as well over the course of the skill acquisition phase. Statistical analyses of invariance indicated significant differences in representation structure between pre-test and post-test (λ < 0.68), indicating furthermore that there was a statistically significant difference between the two groups at the significance level of 5%. Lastly, to evaluate the degree of similarity between the mean dendrogram of the GPG group and the reference dendrogram the ARI was calculated. The ARI analysis revealed that the similarity became higher over the skill acquisition phase. Specifically, ARI_pre_ = 0.51, and ARI_post_ = 0.73 were shown at pre-, and post- retention tests, respectively. Thus, the representation structure of the GPG group approached more of an optimal representation.

#### 3.6.3 Difference between groups

The initial stage of the mental representation structure showed similarity between the groups, as indicated by our invariance analysis (λ = 0.70). Specifically, both the PPG and GPG groups exhibited functional clusters representing down-right, up-right, and down-left movement types during the pre-test.

After 3 days of game practice, the invariance analysis revealed significant differences in the mental representation structure between the two groups (λ = 0.51). This suggests a divergence in the cognitive architecture of the mental representation of the maze game post-practice.

These findings highlight that while the initial mental representations were similar, the subsequent practice led to distinct changes in the mental representation structures of the two groups.

## 4 Discussion

In this paper, we introduce a new novel maze game tool for studying naturalistic motor learning during a bimanual complex motor skill task. We investigated the effects of daily practice by comparing two groups (GPG vs. PPG) on task performance, biomechanics behavior, and tactile pressure. We also examined the relationship between the changes in mental representation structures and the changes in skill performance on a complex movement. In general, we hypothesized that the GPG group would develop more effective outcomes than the PPG group, both in the development of mental representation structure and in improving skill performance, the biomechanics metrics, and the tactile pressure in the early stage of skill learning. Furthermore, we expected changes in mental representation structure to correlate with changes in skill performance and behavior. Our results present new insights into complex bimanual motor learning tasks—a novel maze paradigm—and provide a multi-modal glimpse into how human behavior progresses during early skill acquisition.

### 4.1 Novel sensorimotor learning task

We designed a novel maze paradigm here presented to study bimanual motor learning. Our experimental setup allowed participants to perform unconstrained movements while solving the maze; did not include any artificial go signal, and the duration of each trial did not affect the scoring system, allowing for self-paced movements. Likewise, subjects receive natural somatosensory feedback from the task without unnatural or artificial perturbations that alter the behavior to induce a specific learning strategy. This approach prioritized precision and accuracy as the primary constraints within the maze task, steering participants' learning toward minimizing contact with the maze walls. By not penalizing the time spent on the task, we aimed to encourage careful and deliberate navigation, fostering a blend of rapid movement and strategic control (Kumar et al., 2017). However, the absence of time constraints may have influenced the participants' learning strategies, potentially encouraging more cautious approaches that limited the development of speed-related motor skills (Schmidt et al., 2018). This design choice proved effective in enhancing skill development, as participants refined their techniques over time.

Our results present new insights into complex bimanual motor learning tasks. Our findings demonstrate that our strategy of using precision as the main factor to induce learning was successful. Participants improved in movement and precision, i.e., they learned the novel task without a speed-accuracy trade-off. Performance improvements have been previously observed in simple bimanual tasks (Wolff et al., 1998; Bangert et al., 2010; Loehrer et al., 2016; Kajal et al., 2017) and more complex tasks (Fagard et al., 1985; Mueller et al., 2009; Sisti et al., 2011; Yeganeh Doost et al., 2017; Haar et al., 2020; Schoenfeld et al., 2021) with different induced learning modalities. Therefore, factors such as time, precision, or error can be independently used to observe learning in a novel bimanual motor task.

### 4.2 Task performance and biomechanic

We observed improvement in maze score performance for both groups across the 3 days, supporting the notion that practice leads to the enhancement of motor skills (Ericsson et al., 1993; Ericsson, 2008). This finding aligns with previous research on skill acquisition, which has shown that practice plays a crucial role in developing expertise in various domains (Fitts and Posner, 1967). The more pronounced improvement observed on the third day for both groups suggests that a critical point in skill development might have been reached, where the rate of improvement accelerated. This observation is consistent with the concept of a learning curve (Newell and Rosenbloom, 1980), indicating that participants reached a higher level of familiarity with the maze, leading to the more efficient execution of the task (Magill Richard and Anderson, 2021).

We observed a noticeable improvement during the third day compared to the other days in both groups. Studies investigating motor learning and performance changes across consecutive days of practice have reported mixed results. Typically, significant improvements in performance occur during the first few days of practice, followed by a plateau (Fagard et al., 1985; Karni et al., 1998; Dayan and Cohen, 2011). However, other studies have noted that most significant performance improvements occur at a later stage. Salmoni et al. (1984) reported the most significant improvement occurred between the third and fourth days over 6 days of practice. Overall, various factors like task complexity, individual differences, and type and amount of practice impact the timing and magnitude of performance changes during consecutive days of training on early skilled acquisition tasks (Sánchez et al., 2017; Kantak and Winstein, 2012).

Regarding the difference between the two groups' performance, the good performers' group (GPG) significantly outperformed the poor performers' group (PPG) during each day. The results revealed that GPG maintained a higher level of performance throughout the skill acquisition phase compared to PPG. This finding is consistent with previous studies that have reported that good performers exhibit better retention and transfer of learned motor skills (Shea and Kohl, 1991; Kantak and Winstein, 2012). Several explanations for the differences in performance between good and bad performers have been laid out. One argument is that good performers have more efficient and stable motor control strategies, which allow them to better adapt to changing task demands during learning in contrast to bad performers (Magill and Hall, 1990; Schmidt et al., 2018). Another possible explanation for the differences in performance between good and bad performers during motor learning tasks is related to differences in movement control and joint kinematics. Good performers may have more efficient and effective movement patterns, such as smoother and more coordinated movements, compared to bad performers (Swinnen, 2002; Haaland and Hoff, 2003; Leukel et al., 2012). This can lead to better motor control and precision during motor learning tasks, resulting in faster and more accurate learning. In contrast, bad performers may exhibit less efficient movement patterns, characterized by more variable and uncoordinated movements, which can lead to slower and less accurate learning.

Studies have also shown that good performers have more efficient and precise joint kinematics during movements, allowing them to generate greater movement accuracy and efficiency (Schmidt et al., 2018; Franklin et al., 2012). On the other hand, bad performers may have less efficient and more variable joint kinematics, leading to less precise and less efficient movements. The significant differences in maximum velocity and the number of peaks between the GPG and PPG groups highlight the distinction in motor skill development between the two groups. The GPG group demonstrated better performance, reflecting a more efficient movement strategy and better control of the sphere throughout the maze. This finding aligns with the idea that individuals with higher initial performance levels might have a better ability to adapt and improve their motor skills with practice (Beilock et al., 2002). Research in motor learning has shown that individual differences in factors such as cognitive ability, attentional capacity, and prior experience can impact the rate of skill acquisition (Ackerman, 1987; Wulf et al., 2010). Our results extend this line of research by demonstrating the relationship between initial performance, skill development, and biomechanic progress in a maze navigation task.

### 4.3 Mental representation

With regard to the mental representation structure of the maze game, it was revealed that the mental representation structures of both groups (i.e., good performers and bad performers) changed over time, leading to more elaborate and structured representations in the direction of the expert reference dendrogram as analyzed with the SDA-M (Schack, 2004, 2012), reflecting well the five types of movements identified in the maze (i.e., down-right, right-around, down-left, up-right, right-diagonal-up). This result indicates that in the early stages of skill acquisition in a new motor task, functional changes in task-specific mental representation in long-term memory occur, consistent with the notion that changes in cognitive levels of action organization are linked to changes in the motor level (Frank et al., 2013, 2014, 2016; Kim et al., 2017). In relation to this, Land found a close link between movement kinematics and the structure of expert golfers' cognitive representation of the entire swing movement (Land et al., 2013).

The results of the invariance analysis provide important insights into the mental representation structures of the PPG and GPG groups. Initially, both groups demonstrated similar mental representations, as indicated by the λ value of 0.70. This suggests that participants, regardless of group, started with a comparable cognitive framework for the maze game, characterized by functional clusters for down-right, up-right, and down-left movement types. Accordingly, these clusters are functionally or biomechanically related to movement components and phases for the achievement of action goals (Schack, 2012, 2020). Thus, participants of both groups had a similar initial cognitive architecture of the mental representation of the maze game even though they had no previous performance experience with the task, likely due to common underlying biomechanical principles and functional movement patterns.

However, after 3 days of game practice, the λ value dropped to 0.51, indicating significant differences in the mental representation structures between the two groups. This divergence suggests that practice induced distinct cognitive changes in how each group internalized and represented the maze game. The specific cognitive mechanisms leading to different effects in both groups for the development of mental representation during the early motor learning phase are unclear. A possible explanation is that the slope of representation development is likely to be different due to significant intra-individual top-down mechanism differences present in novices during the early learning stages (Frank et al., 2014; Kim et al., 2017).

The introduction of cognitive primitives in our study intends to connect to broader theories of motor control and learning. These primitives are not only relevant to the specific context of the maze task but also contribute to a deeper understanding of how cognitive and motor processes interact (Schack, 2020; Giszter, 2015). The adoption of cognitive primitives allows us to develop a theoretical framework that can be linked to explicit or implicit tests of motor representation. This framework could provide insights into the cognitive aspects of motor learning that may be applicable to other tasks and contexts (Frank et al., 2014; Schack and Frank, 2021). We see cognitive primitives as analogous to Basic Action Concepts (BACs), which we define as “Basic Movement Concepts” (BMCs). These BMCs encapsulate fundamental units of thought and action planning essential for efficient navigation and task execution.

The introduction of BMCs into our analysis aims to examine how these foundational elements contribute to the overall mental representation and motor learning processes (Schack, 2004; Frank et al., 2016). This approach aligns with previous research highlighting the role of modularity in motor control, where complex movements are composed of simpler, reusable elements (Handzel and Flash, 1999; Giszter, 2015; Langlois et al., 2024). The concept of BMCs thus provides a structured way to analyze the development and refinement of motor skills (Krakauer et al., 2019; Franklin et al., 2012).

We hypothesize that there should be some correlation between low-level sensorimotor actions and higher-level mental representations. Consequently, we assume that the performance or the “learning” in those BACs that resulted differently may also be related to this issue. Future studies should analyze individual performance and the initial representation structure with a detailed focus on specific BACs and investigate the relationship between them for a better understanding of the development of mental representation structure.

Nevertheless, the current results support the notion that task-specific representation structures can be developed through practice, which is in line with the perceptual-cognitive perspective (Mechsner et al., 2001) and the cognitive action architecture approach (CAA; Schack, 2004, 2020), emphasizing the critical role of mental representation for the generation and control of voluntary movements.

### 4.4 Tactile performance

The examination of tactile pressure patterns revealed notable differences between the two groups in relation to skill acquisition and the role of tactile feedback in motor learning. Specifically, the analysis showed significant differences in pressure distribution for the right-hand thumb between the good performers' group (GPG) and the poor performers' group (PPG). The GPG demonstrated a significantly smaller area size on the third day, suggesting more focused pressure application and potentially better control of the maze. This observation might indicate that as skill level increases, participants rely more on refined pressure application for successful navigation (Lederman and Klatzky, 2009; Hatwell et al., 2003). This finding aligns with the concept of force control in motor tasks, where efficient force application is crucial to achieving optimal performance (Enoka, 1997; Lee-Miller et al., 2019), highlighting the importance of tactile feedback in motor learning.

In contrast, the left-hand thumb exhibited no significant differences between the groups, which could imply that this hand played a less critical role in the task's execution, regardless of the group. The absence of significant differences in pressure patterns for the left-hand thumb suggests that the right-hand thumb might play a more dominant role in the maze navigation task. It is important to note that the maze task involved rolling the sphere consistently from the left to the right side, which could contribute to the more significant role of the right hand compared to the left hand during the task execution. This task-specific demand may have led to the observed differences in pressure application patterns between the two hands. Additionally, it is relevant to consider the potential influence of dominant and non-dominant arm reaching control in understanding the differences in pressure patterns (Sainburg and Kalakanis, 2000).

The observed differences between the GPG and PPG groups in our study can be attributed to more efficient movement strategies and better control of the sphere throughout the maze for the GPG group (Krakauer and Mazzoni, 2011; Latash, 2012). Specifically, the enhanced motor control in the GPG group may have allowed them to apply more focused and precise pressure using their right thumb (Naceri et al., 2021). This improved motor control could contribute to their better performance and adaptation in biomechanic velocity and tactile pressure patterns, thereby providing a possible explanation for the distinct outcomes observed between the groups.

Moreover, the observed differences in tactile pressure patterns between the GPG and PPG groups may also be associated with variations in mental representations of the task. The GPG group's more focused and precise pressure application, as evidenced by their right thumb's pressure distribution, suggests that they might have developed a more refined mental representation of the task (Wolpert et al., 2011; Schack, 2004; Schack and Mechsner, 2006; Cienfuegos et al., 2022). This enhanced mental representation could facilitate better motor planning and execution, leading to improved performance and adaptation in biomechanic velocity (Wolpert et al., 2011; Land et al., 2013).

These findings emphasize the role of tactile feedback in motor skill acquisition (Johansson and Flanagan, 2009; Ostry and Gribble, 2016) and provide valuable insights into how pressure distribution changes as training progresses. Understanding the importance of tactile feedback in motor learning can inform the development of more effective training interventions (Wulf and Shea, 2002; Krakauer and Mazzoni, 2011), and help tailor motor learning strategies to the specific needs of individuals.

### 4.5 Overall

Our study on skill acquisition in the context of a novel maze game provides a comprehensive understanding of motor learning by integrating findings from performance, biomechanics, tactile pressure, and mental representation structure analyses. The performance analysis revealed that practice led to enhanced motor skills, with the good performers' group (GPG) consistently outperforming the poor performers' group (PPG). Biomechanic findings indicate that the GPG group demonstrated more efficient movement strategies and better control of the sphere throughout the maze, as reflected in their significant differences in maximum velocity and the lower number of velocity peaks. Mental representation structure analysis showed that both groups developed more elaborate and structured representations over time. The GPG group's post-test representation exhibited a more refined mental representation, potentially contributing to better motor planning and execution. Tactile pressure analysis revealed notable differences in pressure distribution, particularly for the right-hand thumb, with the GPG group demonstrating more focused pressure application and better control. These findings highlight the importance of tactile feedback in motor learning and suggest that the GPG group's improved mental representation and motor control allowed them to apply more focused and precise pressure, leading to better performance and adaptation in biomechanic velocity.

The findings from our study on skill acquisition in the context of a novel maze game contribute significantly to the broader understanding of motor learning and neurocognition. By examining multiple aspects of skill acquisition and performance, including biomechanics, tactile pressure, and mental representation structure, our study provides a more comprehensive understanding of the underlying processes involved in motor learning.

Our results align with the cognitive action architecture approach (CAA; Schack, 2004, 2020) and emphasize the importance of mental representation in the generation and control of voluntary movements (Mechsner et al., 2001; Frank et al., 2013; Cienfuegos et al., 2022). The changes in mental representation structures observed in our study support the notion that practice leads to functional changes in task-specific mental representation in long-term memory (Frank et al., 2014; Kim et al., 2017). This finding is consistent with Schack's CAA approach, which posits that mental representations play a crucial role in motor learning, serving as a foundation for planning and executing motor actions (Schack and Ritter, 2013; Schack and Frank, 2021).

Our study also highlights the importance of examining biomechanics and tactile pressure in motor learning research. By demonstrating significant differences in movement strategies and pressure application between the GPG and PPG groups, our findings emphasize the role of efficient force control and movement patterns in achieving optimal performance (Enoka, 1997; Lee-Miller et al., 2019). These observations align with Meschner's perceptual-cognitive perspective (Mechsner et al., 2001; Schack and Mechsner, 2006), which posits that efficient movement control is crucial for skill acquisition. It highlights the advantages of this approach in adapting actions to effectively function at different levels of motor control.

Furthermore, our findings underline the importance of investigating the interplay between different aspects of skill acquisition, such as biomechanics, tactile pressure, and mental representation structure. Our study shows that the GPG group's more focused pressure application and better control of the sphere in the maze could be related to their more refined mental representation of the task (Wolpert et al., 2011; Land et al., 2013; Schack and Frank, 2021). This observation highlights the need for a holistic approach to motor learning research, considering the intricate relationships between various factors contributing to skill acquisition.

### 4.6 Study limitations

Our study has some limitations that should be acknowledged. The small sample size (*n* = 12) limits the generalizability of our findings. A larger sample size would provide more robust results and reduce the potential for Type I and Type II errors. Additionally, the 3-day training duration may not be sufficient to observe long-term effects on motor learning and mental representation. Grouping participants into good and poor performers based on initial performance could introduce bias, as it assumes that early performance reflects overall improvement potential. Moreover, individual differences in learning ability, such as prior experience, cognitive abilities, and motivation, were not controlled. The results obtained might differ when applied to simpler motor tasks, as the complexity of the maze task may elicit different cognitive and motor processes. Therefore, caution should be exercised when extrapolating these findings to more general contexts. Finally, the absence of time as a performance constraint could have influenced the development of speed-related skills. Future research should address these factors to better understand their impact on motor learning and mental representation.

## 5 Conclusions

In conclusion, our study presents a novel maze game tool for studying naturalistic motor learning during a bimanual complex motor skill task, providing valuable insights into the underlying processes involved in motor learning and neurocognition. By investigating the effects of daily practice on task performance, biomechanics behavior, tactile pressure, and mental representation structure, our study offers a comprehensive understanding of early skill acquisition. The findings demonstrate that efficient force control, movement patterns, and refined mental representations contribute to better performance, highlighting the importance of examining the interplay between these factors.

Our results align with the cognitive action architecture approach and emphasize the significance of mental representation in the generation and control of voluntary movements. The study's findings also underline the importance of investigating biomechanics and tactile pressure in motor learning research, revealing the crucial role of efficient movement control in skill acquisition. Ultimately, our study calls for a holistic approach to motor learning research, considering the intricate relationships between various factors contributing to skill acquisition. This comprehensive understanding of motor learning can pave the way for the development of more effective training interventions and tailored motor learning strategies to meet the specific needs of individuals, further advancing the field of motor learning and neurocognition.

## Data Availability

The raw data supporting the conclusions of this article will be made available by the authors, upon reasonable request.
