# Supplementary material for: Comparative analysis of motor skill acquisition in a novel bimanual task: the role of mental representation and sensorimotor feedback
Source: Front Hum Neurosci. 2024 Sep 11;18:1425090. doi: 10.3389/fnhum.2024.1425090 (PMC11422229; doi:10.3389/fnhum.2024.1425090)
Supplement: Supplementary file 1 [file Data_Sheet_1.pdf]

Supplementary Material

1 SUPPLEMENTARY FIGURES

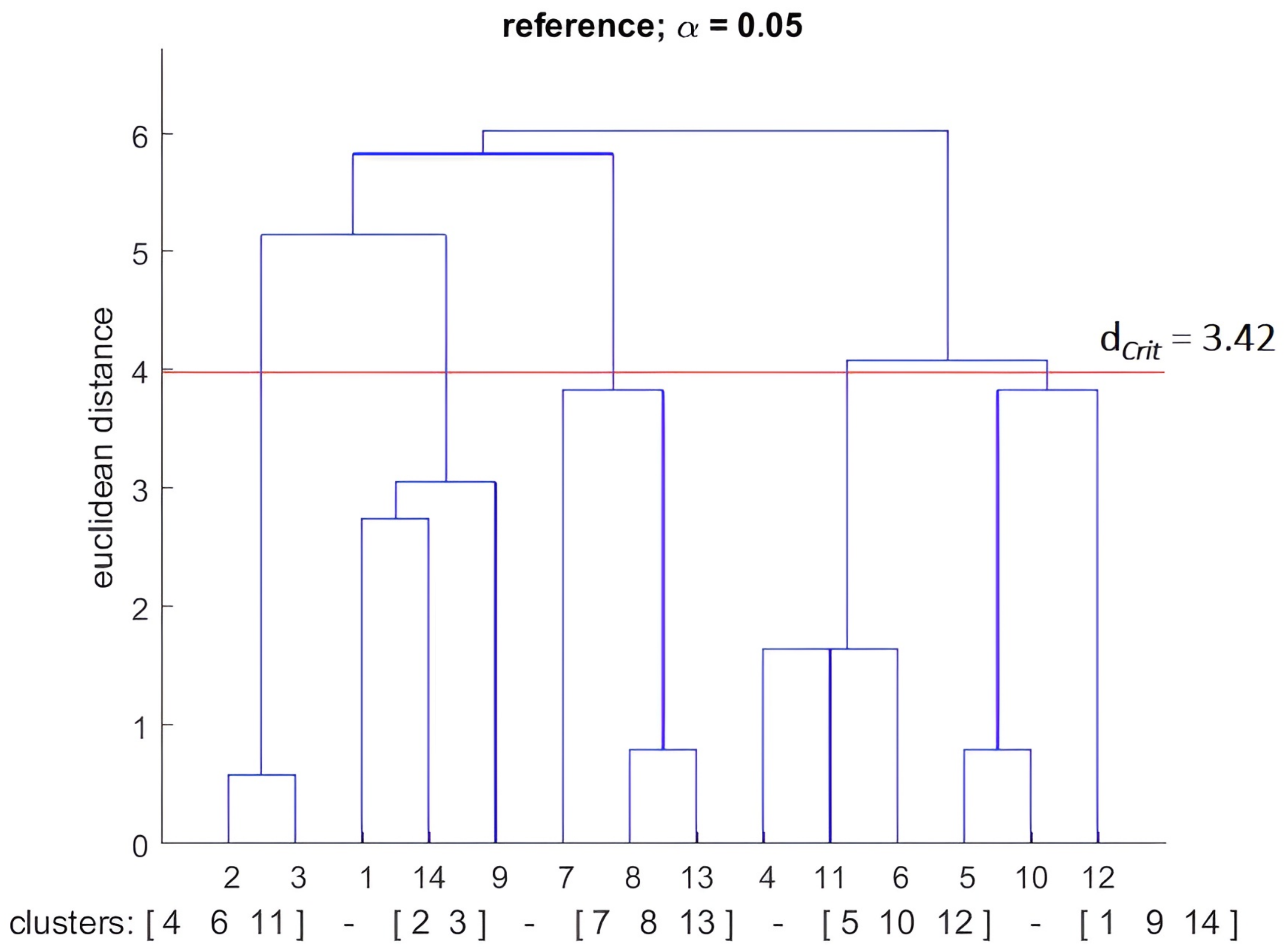

Figure S1. Reference dendrogram indicating the ideal mental representation structure of the maze task. The horizontal line indicates the critical Euclidean distance. The critical value of the Euclidean distance ((d_{Crit} = was 3.42 for an α level of 5%. The basic action concepts (BACs) above this line are considered unrelated. The underlined BACs below this line are considered functionally related to each other.

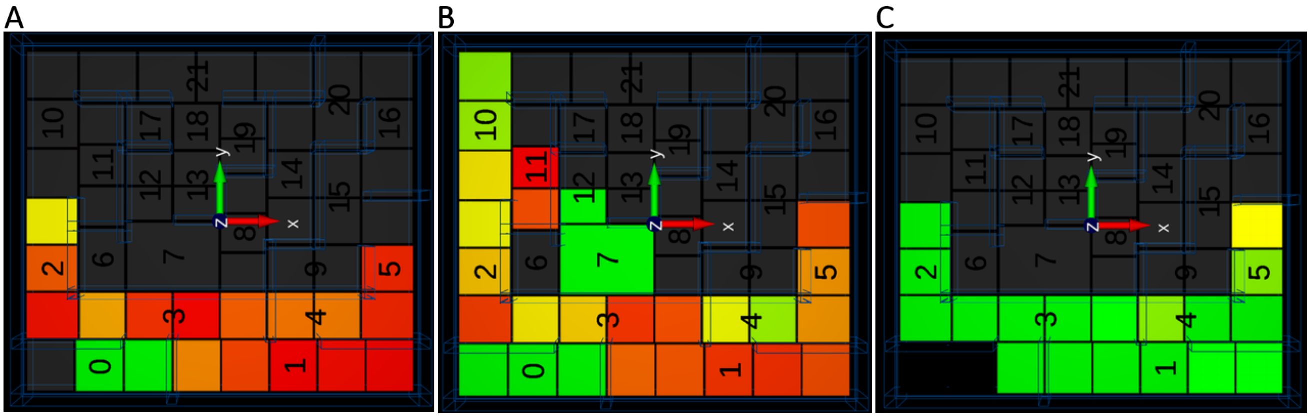

Figure S2. The participant's performance in the maze task is evaluated over three consecutive days. Images (**A**), (**B**), and (**C**) correspond to random trials from day one, day two, and day three, respectively, showing scores based on navigation within the maze. The maze is divided into 21 sections using a computer vision algorithm that scores performance by penalizing wall contacts and evaluating the types of cognitive primitives used. In (**A**), red and orange hues indicate frequent penalized primitives due to wall contact. (**B**) shows a shift toward more 'Guide' primitives, represented by yellow and light green. By (**C**), green sections dominate, indicating near-ideal task execution with minimal penalized primitives, reflecting improved maze navigation. The color-coded metric provides a visual representation of the participant's performance across the three days.
